# Closed-loop motor imagery brain-computer interface-assisted training for upper limb rehabilitation after subacute stroke: clinical and electroencephalographic outcomes from a randomized pilot trial

**DOI:** 10.3389/fneur.2026.1880696

**Published:** 2026-06-24

**Authors:** Wenjie Jin, Xuekang Niu, Yanlin Liu, Zhiwen Zhu, Zhenzhen Gao, Shiyan Wang, Dianhuai Meng, Xinxin Zhu, Haochong Wang, Lingcong Wang, Guangxu Xu, Yajun Mao

**Affiliations:** 1 Rehabilitation Medicine Center, The First Affiliated Hospital of Zhejiang Chinese Medical University (Zhejiang Provincial Hospital of Chinese Medicine), Hangzhou, China; 2 Rehabilitation Medicine Center, The Third Affiliated Hospital of Zhejiang Chinese Medical University, Hangzhou, China; 3 Rehabilitation Medicine Center, Zhejiang Chinese Medical University Affiliated Jiaxing TCM Hospital, Jiaxing, China; 4 Rehabilitation Medicine Center, The First Affiliated Hospital of Nanjing Medical University, Nanjing, China; 5 Rehabilitation Medicine Center, Zhejiang Provincial General Hospital of Chinese People’s Armed Police Forces, Hangzhou, China; 6 School of Life Science and Technology, Institute of Health and Rehabilitation Science, Xi’an Jiaotong University, Xi’an, China

**Keywords:** brain-computer interface, electroencephalography, motor imagery, stroke, upper limb rehabilitation

## Abstract

**Background:**

Closed-loop motor imagery brain-computer interface (MI-BCI) training may support post-stroke upper-limb rehabilitation by coupling motor intention with contingent multisensory feedback. This randomized pilot trial examined its feasibility, safety, short-term clinical effects, and exploratory EEG correlates in patients with subacute stroke.

**Methods:**

In this single-center, assessor-blinded, two-arm pilot trial, 40 patients with first-ever subcortical stroke in the subacute phase were randomized 1:1 to a BCI group or an active control group after a 2-day motor imagery familiarization phase. Both groups received routine medical management, standardized conventional rehabilitation, and dose-matched motor imagery-based hand training for 4 weeks. The BCI group received EEG-contingent closed-loop MI-BCI-assisted training with a soft rehabilitation glove, whereas the control group received non-EEG-contingent glove-assisted motor imagery training under matched training duration, task instructions, device exposure, and multisensory feedback. The primary outcome was the Fugl-Meyer Assessment for the Upper Extremity (FMA-UE). Secondary outcomes included the Action Research Arm Test (ARAT) and Modified Barthel Index (MBI). Exploratory EEG outcomes included FFT%α and FFT%β during motor imagery. Clinical and EEG outcomes were analyzed using baseline-adjusted ANCOVA models, with week-4 values as dependent variables and corresponding baseline values as covariates.

**Results:**

All randomized participants completed the 4-week assessment. In baseline-adjusted ANCOVA models, the BCI group showed higher week-4 scores than the control group for FMA-UE (adjusted mean difference, 13.40 points; 95% CI, 10.71–16.08; *p* < 0.001), ARAT (7.31 points; 95% CI, 4.55–10.07; *p* < 0.001), and MBI (12.21 points; 95% CI, 8.55–15.87; *p* < 0.001). Exploratory EEG analyses also showed higher week-4 FFT%α and FFT%β in the BCI group, with adjusted mean differences of 6.78 percentage points (95% CI, 5.22–8.34; *p* < 0.001) and 3.95 percentage points (95% CI, 2.53–5.36; *p* < 0.001), respectively. No serious adverse events occurred.

**Conclusion:**

Closed-loop MI-BCI-assisted training was feasible and well tolerated in selected patients with subacute stroke. The observed short-term improvements in upper-limb impairment and activity capacity provide preliminary signals of potential benefit beyond dose-matched non-EEG-contingent feedback training. Exploratory EEG findings suggest task-related modulation of alpha- and beta-band sensorimotor rhythmic activity, but should be interpreted as hypothesis-generating rather than confirmatory evidence of neural reorganization. Larger multicenter trials with longer follow-up, rigorous neurophysiological analyses, and real-world upper-limb use outcomes are needed.

**Clinical Trial Registration:**

ChiCTR2400083992. https://www.chictr.org.cn/showproj.html?proj=229529

## 1 Introduction

Stroke is an acute neurological injury caused by vascular occlusion or rupture, resulting in focal ischemia and hypoxia, and remains one of the leading causes of death and disability worldwide (1). Post-stroke upper limb motor impairment is a major barrier to the recovery of independent living and social participation (2, 3). The subacute phase is generally regarded as a period of heightened neuroplasticity and responsiveness to rehabilitation; however, recovery trajectories vary substantially across patients (4, 5). A clinically observed pattern is that global upper limb impairment, proximal control, and movement initiation may improve earlier than dexterous hand function (6). Recovery of grasping, pinching, and object manipulation is often more limited because these tasks require distal motor control, sensory feedback, and coordinated task execution. Accordingly, post-stroke upper limb rehabilitation studies should assess outcomes at both the impairment level, such as FMA-UE, and the activity level, such as ARAT, to better determine the clinical relevance of an intervention (7).

Under conventional stroke rehabilitation, some degree of functional improvement is often observed over the short term in patients in the subacute stage. However, because spontaneous recovery, treatment dose, and training content vary considerably, clinical trials aiming to identify the incremental value of emerging technologies require more rigorous control conditions and more cautious interpretive frameworks. In recent years, advances in medical technology and increased interdisciplinary collaboration have accelerated the development of artificial intelligence-based approaches in medicine, some of which have shown encouraging potential. Among these, brain-computer interface (BCI) technology has emerged as one of the most representative and promising approaches (8). An increasing number of studies have investigated the clinical efficacy and potential mechanisms of BCI-based interventions for neurological disorders. These systems enable users to control external devices or receive feedback based on their brain activity patterns, thereby offering a novel strategy for stroke rehabilitation. By harnessing neuroplasticity, BCI systems are intended to facilitate motor recovery through activation of neural circuits involved in motor planning and execution (9).

Motor imagery-based brain-computer interface training provides an intention-driven, closed-loop rehabilitation strategy for patients with limited voluntary movement (10). By decoding motor imagery or attempted movement and delivering temporally contingent feedback through visual or auditory cues, functional electrical stimulation, virtual reality, or robotic assistance, this approach may strengthen sensorimotor integration, enhance training engagement, and promote activity-dependent neuroplasticity (11, 12). Although the existing literature generally suggests that such approaches are promising, substantial heterogeneity remains across studies in terms of stroke stage, decoding strategy, feedback modality, training dose, control condition, and selected outcome measures. In addition, some trials have been limited by small sample sizes, insufficient follow-up, or dose mismatch, making it difficult to distinguish the incremental value of the closed-loop BCI component itself from differences in devices or training intensity. Against this background, we conducted an assessor-blinded randomized controlled trial to compare a multimodal closed-loop motor imagery BCI rehabilitation package with a dose-matched active control intervention. Feasibility outcomes and exploratory neurophysiological correlates were also recorded to provide preliminary effect estimates and inform the design of future confirmatory trials.

## 2 Materials and methods

### 2.1 Study design and ethics

This study was designed as a single-center, assessor-blinded, two-arm, parallel-group randomized pilot trial. Eligible participants were randomly assigned in a 1:1 ratio to receive either the BCI group or the control group. The intervention lasted 4 weeks, with outcome assessments performed at baseline before the first intervention session and at the end of week 4.

The study was approved by the Zhejiang Chinese Medical University Affiliated Jiaxing TCM Hospital ethics committee (SL-2024-0014) and was registered in the Chinese Clinical Trial Registry (ChiCTR2400083992). Written informed consent was obtained from all participants before enrolment. The trial was conducted and reported in accordance with the CONSORT 2010 extension for randomized pilot and feasibility trials (13).

### 2.2 Participants

The research subjects were derived from patients with subacute stroke who received inpatient rehabilitation treatment at the Rehabilitation Medicine Center of the First Affiliated Hospital of Zhejiang Chinese Medical University. The inclusion criteria were as follows: (1) age 16–80 years; (2) right-handedness; (3) first-ever subcortical stroke confirmed by neuroimaging; (4) clinically stable condition, 2 weeks to 3 months after stroke onset; (5) Brunnstrom stage II-IV of the affected hand; (6) Mini-Mental State Examination score >26, with normal or corrected-to-normal vision; and (7) ability to understand and attempt the motor imagery task involving grasping movements of the affected hand and provide written informed consent.

The exclusion criteria were as follows: (1) cortical stroke or multiple stroke; (2) severe spasticity of the affected upper limb, defined as a Modified Ashworth Scale score >2, or marked pain, defined as a visual analog scale score >4; (3) history of epilepsy, a first-degree relative with idiopathic epilepsy, or previous use of antiepileptic medication; (4) cardiac pacemaker, intracranial metal implants, skull defects, or other contraindications to neurophysiological assessment or training; and (5) severe attentional, visual, language, or communication impairment that would preclude safe participation or reliable task completion.

### 2.3 Run-in phase

Before randomization, candidates who met the screening criteria underwent a 2-day motor imagery familiarization phase. This run-in phase was used to ensure that participants could understand the motor imagery instructions, attempt the affected-hand grasping imagery task, and tolerate the training procedures. Candidates who were unable to complete this familiarization phase were not randomized. This design was intended to improve the feasibility and safety of training delivery. However, it may have enriched the randomized sample with participants who were more capable of performing motor imagery tasks (14).

### 2.4 Sample size rationale

As this was a randomized pilot trial, a formal efficacy-based sample size calculation was not performed. A convenience sample of 40 participants was planned, with 20 participants in each group. The main purposes of this sample size were to estimate the variability and preliminary effect sizes of the clinical outcomes, evaluate trial completion and safety, and inform sample size calculation and protocol optimization for a future adequately powered confirmatory trial.

### 2.5 Randomization and allocation concealment

After completion of the run-in phase and confirmation of eligibility, participants were randomly allocated to the BCI group or control group in a 1:1 ratio. The randomization sequence was generated by an independent researcher who was not involved in intervention delivery or outcome assessment, using a computer-generated allocation schedule. Allocation concealment was maintained using sequentially numbered, opaque, sealed envelopes. Group assignment was performed by the study coordinator after enrolment and baseline assessment.

### 2.6 Blinding

Outcome assessors were blinded to group allocation throughout the study. Because of the nature of the intervention and differences in device control mode, therapists and participants could not be fully blinded. To reduce detection and information bias, intervention delivery and outcome assessment were performed by different personnel. Participants were also instructed not to disclose their group assignment to the assessors during the evaluation sessions. To reduce differential expectancy and motivational effects, both groups wore the same EEG cap and soft robotic glove and received matched session duration, therapist contact, task instructions, cueing structure, and multisensory feedback. However, therapists and participants could not be fully blinded to the device control mode, and formal treatment-credibility or expectancy ratings were not collected.

### 2.7 Interventions

Both groups received routine medical management, standardized conventional rehabilitation, and dose-matched motor imagery-based hand training. Conventional rehabilitation included physical therapy, occupational therapy, and exercise therapy, delivered for 30 min per session, once daily, 5 days per week, for 4 weeks. Motor imagery-based hand training was delivered for 20 min per session, once daily, 5 days per week, for 4 weeks. In both groups, the training task primarily involved imagining grasping movements with the affected hand, and the training duration, task instructions, and cueing structure were matched between groups.

**Figure 1:**
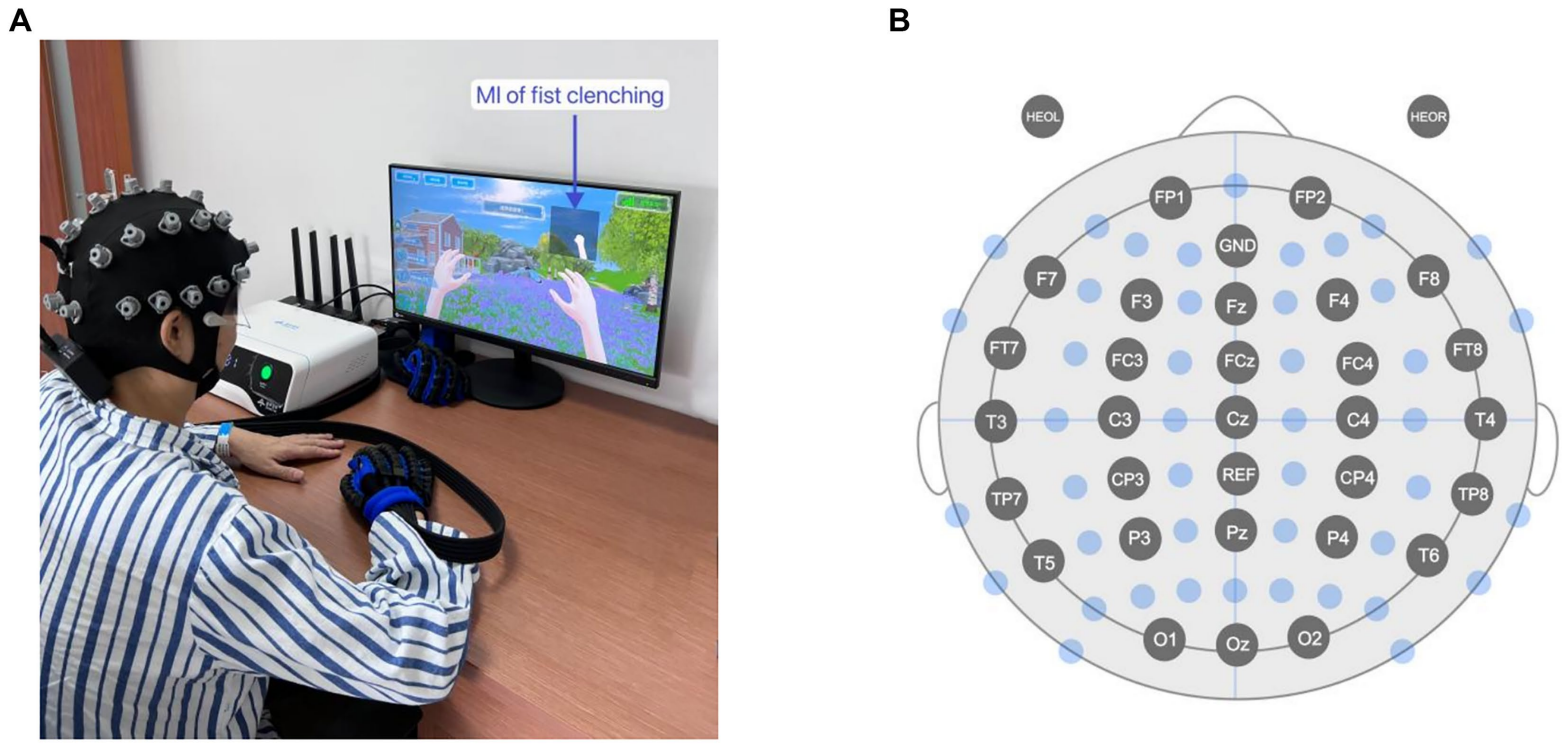
**(A)** Shows the structure of the closed-loop feedback MI-BCI rehabilitation training system, and **(B)** shows the electroencephalography (EEG) channel montage used for signal acquisition.

The multimodal closed-loop motor imagery BCI rehabilitation system consisted of an EEG acquisition and online analysis module, a soft hand rehabilitation robotic glove, and a virtual reality-based feedback module. The system components and the interface of the virtual reality training system are shown in Figure 1A. During training, scalp EEG signals were acquired using a 32-channel saline-based EEG system (ZhenTec NT1, Xi’an Feima Intelligent Technology Co., Ltd., Xi’an, China) at a sampling rate of 500 Hz. Electrode impedance was maintained at ≤20 kΩ before recording. The online analysis module calculated an individualized EEG engagement/control index during the motor imagery task and determined whether the robotic glove should be triggered.

In the BCI group, participants received closed-loop MI-BCI-assisted training. At the beginning of each session, participants wore the EEG cap and the soft rehabilitation glove, and the therapist explained the training procedure and key instructions. The virtual reality training program was then started for cyclic visual motor imagery feedback training. Each training trial consisted of a 2-s visual cue period, a 4-s motor imagery period, a 4-s feedback period, and a 2-s rest period. During the visual cue period, participants were instructed to prepare for affected-hand grasping imagery. During the motor imagery period, participants imagined grasping movements with the affected hand without overt voluntary movement, while scalp EEG signals were recorded and analyzed online. When the EEG engagement/control index exceeded the predefined threshold, the soft robotic glove provided assisted grasping movement accompanied by visual, auditory, and tactile feedback. When the threshold was not reached, the robotic glove was not triggered, and encouraging auditory feedback was provided.

In the control group, participants received dose-matched motor imagery training with a non-EEG-contingent soft rehabilitation glove. Participants wore the same EEG cap and soft rehabilitation glove to match the wearing experience and device exposure; however, the EEG signals were not used to control the robotic glove. Under the same training schedule, task instructions, and cueing structure, participants performed affected-hand grasping motor imagery and received preprogrammed, non-EEG-triggered robotic glove assistance with corresponding visual, auditory, and tactile feedback. Thus, the active control condition was designed to match treatment duration, therapist contact, motor imagery instructions, device exposure, and multisensory feedback as closely as possible, while removing the closed-loop EEG-contingent control component. The active control condition was designed to maximize face validity and treatment credibility by matching equipment exposure, therapist attention, cueing structure, and multisensory feedback while removing only the real-time EEG-contingent triggering component.

### 2.8 Outcome measures

All outcomes were assessed at baseline (week 0, before the first intervention session) and again at the end of week 4 by trained assessors blinded to group allocation. To minimize detection bias, outcome assessment and treatment delivery were performed by different study personnel, and participants were reminded not to disclose their allocation during evaluation. The primary outcome was the Fugl-Meyer Assessment for the Upper Extremity (FMA-UE), a stroke-specific measure of upper-limb motor impairment originally developed for post-stroke sensorimotor evaluation. The FMA-UE contains 33 items scored on a 3-point ordinal scale (0–2), yielding a total score from 0 to 66, with higher scores indicating less motor impairment. The FMA-UE was chosen as the primary endpoint because it is one of the most frequently used and best-validated upper-limb impairment measures in stroke rehabilitation research, with excellent inter-rater reliability after standardized assessor training (15).

The secondary clinical outcomes were the Action Research Arm Test (ARAT) and the Modified Barthel Index (MBI). The ARAT is an activity-level measure of upper-limb performance comprising 19 items across four subdomains (grasp., grip, pinch, and gross movement), scored from 0 to 3 for a total score of 0 to 57, with higher scores indicating better upper-limb activity capacity and dexterity. The ARAT has demonstrated excellent inter-rater reliability and good construct validity in people with stroke (16). The MBI was used to assess independence in basic activities of daily living. In this manuscript, the MBI total score ranges from 0 to 100, with higher scores indicating greater independence (17). The neurophysiological outcomes were prespecified FFT%α and FFT%β during the motor-imagery task. These variables were selected on the basis of prior motor-imagery/MI-BCI literature and their theoretical relevance to alpha−/mu- and beta-band sensorimotor rhythm modulation during motor imagery and movement preparation. FFT%α and FFT%β were defined as relative spectral power percentages of the α and β bands, respectively, calculated from artifact-free sensorimotor EEG epochs using fast Fourier transform (18). Because these measures reflect relative spectral power rather than baseline-normalized ERD/ERS, they were treated as exploratory indices of task-related rhythmic modulation rather than direct measures of cortical efficiency or neuroplastic reorganization.

### 2.9 EEG acquisition and preprocessing

EEG signals were recorded during the motor imagery task using the same 32-channel saline-based EEG system (ZhenTec NT1, Xi’an Feima Intelligent Technology Co., Ltd., Xi’an, China) at a sampling rate of 500 Hz. Electrode impedance was maintained at ≤20 kΩ before recording. Recording electrodes were positioned according to the international 10–20 system, covering frontal, central, frontocentral, centroparietal, parietal, occipital, and sensorimotor-related regions. The recorded channels included C1, C2, C3, C4, C5, C6, CP1, CP2, CP3, CP4, CP5, CP6, CPz, Cz, F3, F4, FC3, FC4, FC5, FC6, FCz, Fz, O1, O2, P1, P2, P3, P4, PO3, PO4, POz, and Pz. The reference electrode was placed on the earlobe, and the ground electrode was placed at Fpz, as shown in Figure 1B.

EEG data were preprocessed offline using MATLAB R2023b (MathWorks, Natick, MA, United States). Raw EEG data were visually inspected to identify and exclude segments contaminated by excessive movement or physiological artifacts. The EEG signals were filtered using a 5–30 Hz finite impulse response band-pass filter and a 50 Hz notch filter to reduce power-line noise. Artifact rejection and data segmentation were subsequently performed. EEG epochs corresponding to the 4-s motor imagery period were extracted from the continuous EEG recordings. Baseline correction was performed using the 1-s pre-imagery period immediately preceding each motor imagery epoch.

Artifact-free sensorimotor EEG epochs were used for fast Fourier transform analysis. FFT%α and FFT%β were calculated as the relative power percentages of the predefined α and β frequency bands within the reference spectrum. These EEG outcomes were interpreted as exploratory indices of sensorimotor rhythmic modulation during motor imagery rather than direct measures of event-related desynchronization or synchronization (19).

### 2.10 Statistical analysis

All statistical analyses were conducted in the post-randomization population, which served as the primary analysis set. Because no participant was lost to follow-up and no post-randomization outcome data were missing, the full analysis set and per-protocol analysis set were identical. Continuous variables are presented as mean ± standard deviation, and categorical variables as number and percentage. Baseline demographic and clinical characteristics were summarized descriptively, without significance testing between groups.

Feasibility outcomes, including recruitment, retention, adherence, usable EEG-session rate, and adverse events, were analyzed descriptively. The primary clinical outcome was the Fugl-Meyer Assessment for the Upper Extremity (FMA-UE). Secondary outcomes included the Action Research Arm Test (ARAT) and Modified Barthel Index (MBI). Exploratory neurophysiological outcomes included FFT%α and FFT%β during the motor-imagery task. FFT%α and FFT%β were calculated as relative spectral power proportions and multiplied by 100 for reporting as percentages.

For each clinical and neurophysiological outcome, the primary inferential analysis used analysis of covariance (ANCOVA), with the week-4 value as the dependent variable, treatment group as a fixed effect, and the corresponding baseline value as a covariate. Baseline-adjusted mean differences at week 4 were calculated as BCI minus control and are reported with standard errors, 95% confidence intervals, *p* values, and partial η^2^ as the effect size. Baseline-by-group interactions were examined to assess the homogeneity-of-regression-slopes assumption and were not retained in the final models when not statistically significant. Raw change scores, calculated as week-4 minus baseline values, are presented descriptively. Within-group paired-sample t tests and unadjusted between-group comparisons of change scores were considered supportive analyses only. For FFT%α and FFT%β, adjusted mean differences are expressed in percentage points.

The distribution of continuous variables and model residuals was assessed by visual inspection and normality testing. Given the pilot nature of this study, inference focused on effect estimates, 95% confidence intervals, and direction of change rather than definitive hypothesis testing. No formal adjustment for multiple comparisons was applied to exploratory neurophysiological outcomes; therefore, these analyses should be interpreted as hypothesis-generating. A two-sided nominal *p* value < 0.05 was considered statistically significant. Analyses were performed using SPSS version 26.0 (IBM Corp., Armonk, NY, United States).

## 3 Results

### 3.1 Participant flow and baseline characteristics

**Figure 2:**
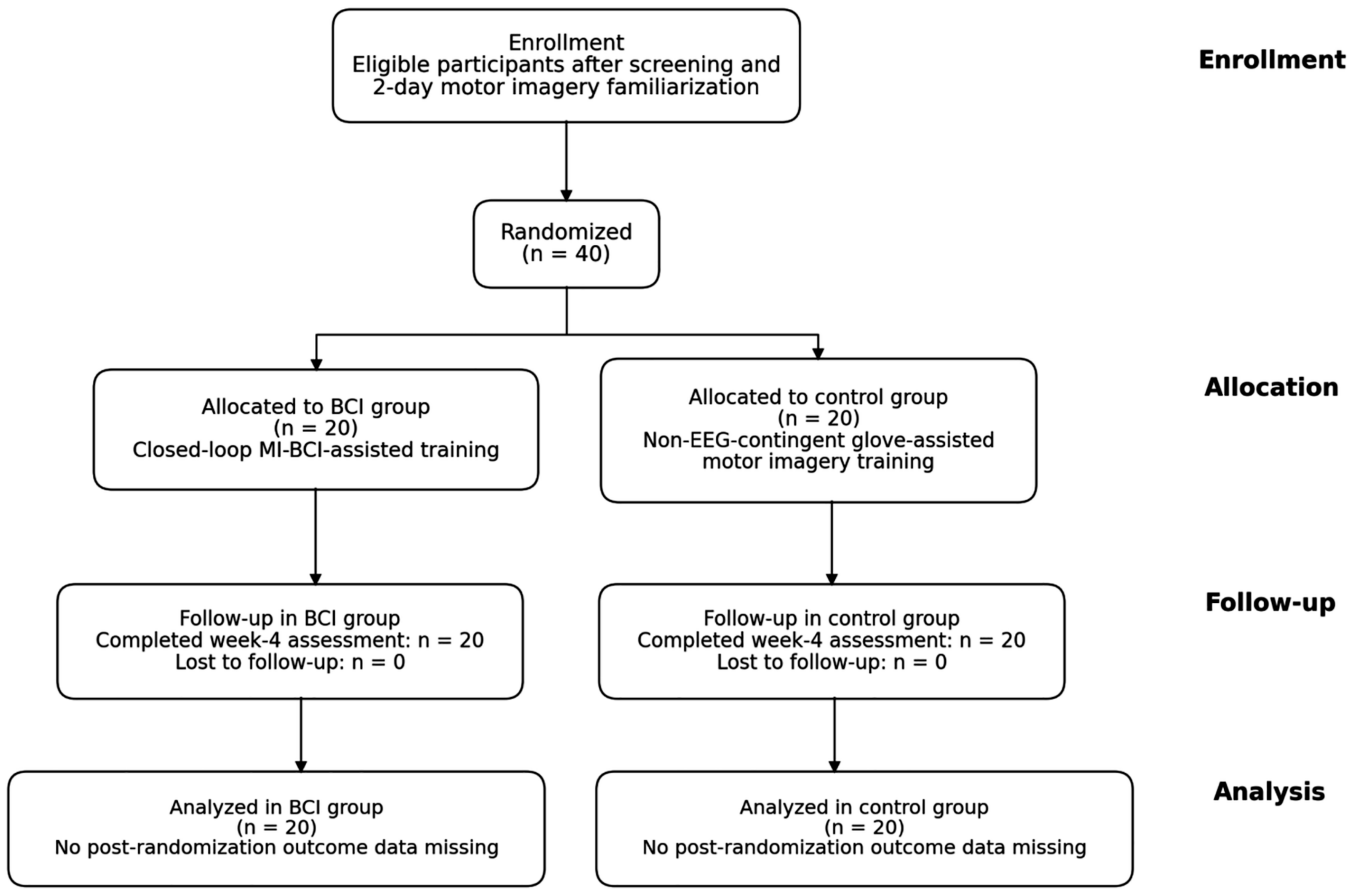
CONSORT-style participant flow diagram. Forty eligible participants were randomized after screening and completion of the 2-day motor imagery familiarization phase, with 20 assigned to the BCI group and 20 assigned to the control group. No randomized participant was lost to follow-up, all participants completed the week-4 assessment, and all randomized participants were included in the analysis.

Participant flow and analysis populations. A CONSORT-style participant flow diagram is shown in Figure 2. Forty participants were randomized after completion of screening and the 2-day motor imagery familiarization phase, with 20 assigned to the BCI group and 20 to the control group. No randomized participant was lost to follow-up, all participants completed the week-4 assessment, and no post-randomization outcome data were missing. Accordingly, the intention-to-treat/full analysis set and the per-protocol analysis set were identical (*n* = 40). Mean age was 53.80 ± 11.15 years in the BCI group and 52.00 ± 11.88 years in the control group; the proportions of male participants were 70.0 and 80.0%, respectively. Mean time since stroke onset was 35.70 ± 16.32 days in the BCI group and 32.90 ± 19.54 days in the control group. All participants had first-ever subcortical stroke, including subcortical ischemic infarction or subcortical intracerebral hemorrhage confirmed by neuroimaging. In line with CONSORT guidance, no significance tests were performed for baseline comparisons. Baseline characteristics are presented descriptively in Table 1.

**Table 1:** Baseline characteristics of the sample.

Characteristic	Control group (*n* = 20)	BCI group (*n* = 20)
Age, years	52.00 ± 11.88	53.80 ± 11.15
Male, *n* (%)	16 (80.0)	14 (70.0)
Education, years	6.95 ± 4.46	8.10 ± 5.61
Stroke type, ischemic/hemorrhagic, *n*	12/8	12/8
Affected hemisphere, left/right, *n*	10/10	8/12
Time since stroke onset, days	32.90 ± 19.54	35.70 ± 16.32
Hand Brunnstrom stage, mean ± SD	2.50 ± 0.85	2.90 ± 0.99
Lesion volume, ml	30.3 ± 18.2	28.8 ± 16.8

Values are mean ± SD or *n* (%).

### 3.2 Clinical outcomes

#### 3.2.1 Primary outcome

FMA-UE improved in both groups over 4 weeks. The BCI group increased from 15.20 ± 13.53 to 35.00 ± 16.42 points, corresponding to a raw mean change of 19.80 ± 5.85 points, whereas the control group increased from 16.90 ± 16.93 to 23.40 ± 17.02 points, corresponding to a raw mean change of 6.50 ± 1.18 points. In the baseline-adjusted ANCOVA model, the BCI group showed higher week-4 FMA-UE scores than the control group, with an adjusted mean difference of 13.40 points (BCI minus control; SE, 1.33; 95% CI, 10.71 to 16.08; *p* < 0.001; partial η^2^ = 0.734).

#### 3.2.2 Secondary clinical outcomes

ARAT also improved in both groups. The BCI group improved from 5.80 ± 15.16 to 18.65 ± 14.05 points, with a raw mean change of 12.85 ± 5.90 points, whereas the control group improved from 5.70 ± 15.35 to 11.25 ± 14.99 points, with a raw mean change of 5.55 ± 2.24 points. In baseline-adjusted ANCOVA, the adjusted mean difference at week 4 was 7.31 points in favor of the BCI group (SE, 1.36; 95% CI, 4.55 to 10.07; *p* < 0.001; partial η^2^ = 0.438).

For MBI, the BCI group increased from 50.80 ± 12.47 to 79.50 ± 10.44 points, corresponding to a raw mean change of 28.70 ± 7.04 points, whereas the control group increased from 48.30 ± 18.16 to 65.40 ± 15.20 points, corresponding to a raw mean change of 17.10 ± 6.52 points. The baseline-adjusted ANCOVA model showed a higher week-4 MBI score in the BCI group than in the control group, with an adjusted mean difference of 12.21 points (SE, 1.81; 95% CI, 8.55 to 15.87; *p* < 0.001; partial η^2^ = 0.553). Clinical outcomes are presented descriptively and with baseline-adjusted ANCOVA estimates in Table 2.

**Table 2:** Comparison of clinical outcomes between the control and BCI groups before and after intervention, with baseline-adjusted ANCOVA estimates at week 4.

Outcomes	Control group			BCI group			Baseline-adjusted mean difference at week 4, BCI minus control	SE	95% CI	*p* value	Partial η^2^
	Baseline	Week 4	Control change	Baseline	Week 4	BCI change					
FMA-UE	16.90 ± 16.93	23.40 ± 17.02	6.50 ± 1.18	15.20 ± 13.53	35.00 ± 16.42	19.80 ± 5.85	13.40	1.33	10.71–16.08	<0.001	0.734
ARAT	5.70 ± 15.35	11.25 ± 14.99	5.55 ± 2.24	5.80 ± 15.16	18.65 ± 14.05	12.85 ± 5.90	7.31	1.36	4.55–10.07	<0.001	0.438
MBI	48.30 ± 18.16	65.40 ± 15.20	17.10 ± 6.52	50.80 ± 12.47	79.50 ± 10.44	28.70 ± 7.04	12.21	1.81	8.55–15.87	<0.001	0.553

Values for baseline, week 4, and raw change are mean ± SD and are presented descriptively. The primary inferential analysis used analysis of covariance (ANCOVA), with the week-4 score as the dependent variable, group as a fixed effect, and the baseline score of the same outcome as a covariate. The baseline-adjusted mean difference is expressed as BCI minus control. Group-by-baseline interaction terms were examined and were not retained in the final models because they were not statistically significant. Partial η^2^ is reported as the effect size.

### 3.3 Exploratory neurophysiological outcomes

FFT%α and FFT%β were reported as percentages after multiplying the relative spectral power proportions by 100. For FFT%α, the BCI group increased from 4.27 ± 2.84% at baseline to 7.89 ± 2.85% at week 4, corresponding to a raw mean change of 3.62 ± 4.31 percentage points. In contrast, the control group changed from 1.78 ± 0.44% to 1.46 ± 0.52%, corresponding to a raw mean change of −0.32 ± 0.62 percentage points. In baseline-adjusted ANCOVA, the BCI group showed higher week-4 FFT%α than the control group, with an adjusted mean difference of 6.78 percentage points (BCI minus control; SE, 0.77; 95% CI, 5.22 to 8.34; *p* < 0.001; partial η^2^ = 0.678).

For FFT%β, the BCI group increased from 2.30 ± 1.07% to 4.95 ± 1.99%, corresponding to a raw mean change of 2.64 ± 2.16 percentage points, whereas the control group changed from 0.54 ± 0.20% to 0.70 ± 0.23%, corresponding to a raw mean change of 0.16 ± 0.34 percentage points. The baseline-adjusted ANCOVA model showed higher week-4 FFT%β in the BCI group than in the control group, with an adjusted mean difference of 3.95 percentage points (SE, 0.70; 95% CI, 2.53 to 5.36; *p* < 0.001; partial η^2^ = 0.463). Exploratory neurophysiological outcomes are presented descriptively and with baseline-adjusted ANCOVA estimates in Table 3.

**Table 3:** Comparison of exploratory neurophysiological outcomes between the control and BCI groups before and after intervention, with baseline-adjusted ANCOVA estimates at week 4.

Outcomes	Control group			BCI group			Baseline-adjusted mean difference at week 4, BCI minus control	SE	95% CI	*p* value	Partial η^2^
	Baseline	Week 4	Control change	Baseline	Week 4	BCI change					
FFT%α, %	1.78 ± 0.44	1.46 ± 0.52	−0.320 ± 0.62	4.27 ± 2.84	7.89 ± 2.85	3.62 ± 4.31	6.78	0.77	5.22 to 8.34	<0.001	0.678
FFT%β, %	0.54 ± 0.20	0.70 ± 0.23	0.16 ± 0.34	2.30 ± 1.07	4.95 ± 1.99	2.64 ± 2.16	3.95	0.70	2.53 to 5.36	<0.001	0.463

Values for baseline, week 4, and raw change are mean ± SD and are presented descriptively. FFT%α and FFT%β are reported as percentages after multiplying the relative spectral power proportions by 100. The primary inferential analysis used analysis of covariance (ANCOVA), with the week-4 value as the dependent variable, group as a fixed effect, and the baseline value of the same outcome as a covariate. The baseline-adjusted mean difference is expressed as BCI minus control and is reported in percentage points. SE indicates standard error; CI, confidence interval. Partial η^2^ is reported as the effect size and should be interpreted cautiously because of the pilot sample size. Baseline-by-group interaction terms were examined and were not retained in the final models because they were not statistically significant.

These exploratory EEG findings suggest greater task-related modulation of alpha- and beta-band relative spectral activity during motor imagery in the BCI group. However, because FFT%α and FFT%β are relative spectral power indices, these findings should be interpreted as hypothesis-generating neurophysiological signals rather than direct evidence of improved cortical efficiency or confirmed neural reorganization.

No serious adverse events occurred during the intervention period. Mild transient fatigue was reported by 1/20 participants in the BCI group and 2/20 participants in the control group. Mild transient scalp or hand-skin discomfort was reported by 2/20 and 2/20 participants, respectively. No adverse event led to treatment discontinuation, and all symptoms resolved spontaneously or after brief equipment adjustment without specific treatment.

**Figure 3:**
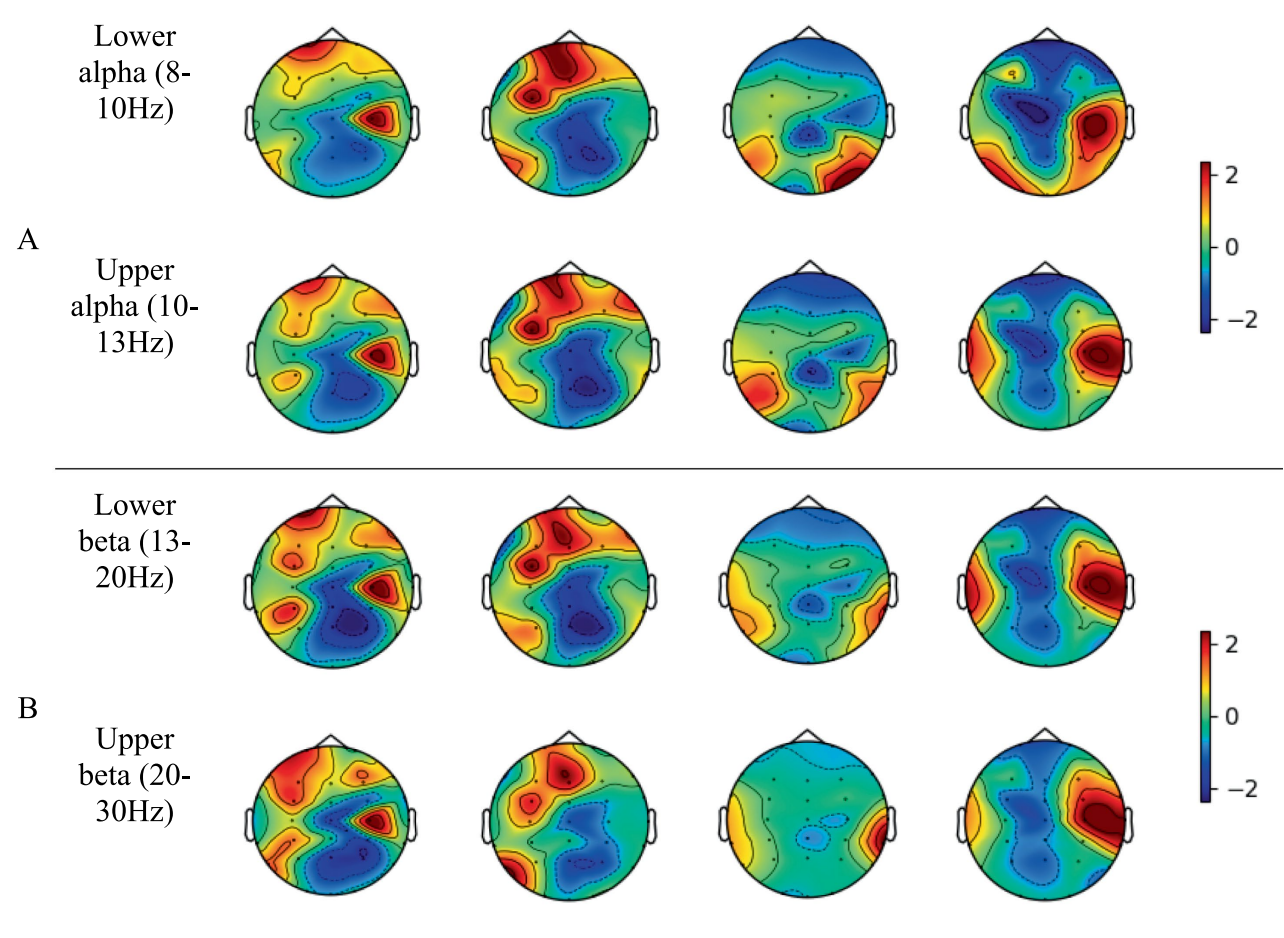
Descriptive scalp topographic maps of alpha- and beta-band EEG activity during the motor imagery task. **A** shows lower- and upper-alpha distributions, and **B** shows lower- and upper-beta distributions, for the control and BCI groups at baseline and week 4. These maps are provided for visualization only. Because no channel-wise or voxel-wise statistical topographic analysis was performed, apparent spatial differences should not be interpreted as confirmed region-specific cortical modulation. The predefined neurophysiological outcomes were FFT%α and FFT%β.

Scalp topographic maps were used to provide descriptive visualization of frequency-specific EEG activity during the motor imagery task. As shown in Figure 3, the BCI group appeared to show more visually evident changes in alpha- and beta-band activity after 4 weeks of intervention than the control group. However, these maps were interpreted descriptively only, because no channel-wise or topographic statistical analysis was performed. Therefore, the topographic findings should not be interpreted as evidence of statistically confirmed region-specific cortical modulation.

## 4 Discussion

This assessor-blinded randomized pilot trial investigated the effects of closed-loop motor imagery brain-computer interface (MI-BCI)-assisted training on upper limb rehabilitation in patients with subacute stroke. After 4 weeks of intervention, participants in the BCI group showed greater improvements in upper limb motor impairment and activity performance, as reflected by FMA-UE and ARAT, than those in the dose-matched active control group. A greater improvement in MBI was also observed in the BCI group, although this finding should be interpreted as supportive rather than specific evidence of upper limb functional recovery. Exploratory EEG analyses further showed more pronounced changes in FFT%α and FFT%β during the motor imagery task in the BCI group. No serious adverse events occurred. Taken together, these findings suggest that closed-loop MI-BCI-assisted training is feasible in a selected subacute stroke population and may provide preliminary short-term benefits for upper limb motor recovery beyond those achieved by dose-matched motor imagery training with non-EEG-contingent feedback.

The present findings are generally consistent with recent clinical evidence suggesting that BCI-based and MI-BCI-based rehabilitation may improve upper limb motor outcomes after stroke (20, 21). A recent randomized controlled trial showed that BCI rehabilitation training, when added to conventional rehabilitation, further improved upper limb motor function in patients with ischemic stroke (22). Other recent studies have also reported that MI-BCI rehabilitation may enhance upper limb performance and cortical activation, and that MI-contingent BCI feedback may produce greater functional and neuroplasticity-related effects than MI-independent feedback (10, 23). The present study extends this line of evidence by focusing on patients in the subacute stage and by comparing closed-loop EEG-contingent training with an active control condition matched for motor imagery practice, robotic exposure, and multisensory feedback.

The interpretation of these findings should consider the biological and clinical characteristics of the subacute phase after stroke. This period is commonly regarded as a window of heightened neuroplasticity and responsiveness to rehabilitation, but spontaneous neurological recovery and rehabilitation-induced plasticity occur simultaneously (24). In addition, recovery trajectories vary considerably across patients, depending on baseline impairment, lesion characteristics, corticospinal tract integrity, and individual capacity for motor relearning (25, 26). Therefore, the improvements observed in both groups are not unexpected. Importantly, the control group in this study did not receive usual care alone, but received dose-matched motor imagery training combined with non-EEG-contingent soft rehabilitation glove. This active control design helped reduce potential confounding effects related to treatment duration, therapist contact, task instruction, device exposure, and multisensory stimulation. Under this design, the larger gains observed in the BCI group may be more plausibly attributed to the additional EEG-contingent closed-loop component. Nevertheless, because this was a pilot trial, the observed between-group differences should be regarded as preliminary efficacy signals rather than definitive evidence of treatment superiority.

The concurrent improvements in FMA-UE and ARAT provide clinically relevant information from complementary perspectives. FMA-UE primarily reflects impairment-level motor recovery, including abnormal synergy, selective motor control, and proximal-to-distal movement recovery. ARAT, in contrast, evaluates activity-level upper limb performance, including grasping, gripping, pinching, and gross movement. This multidimensional assessment strategy is consistent with recommendations that stroke rehabilitation trials should evaluate sensorimotor recovery across both body function and activity domains (6, 7, 16, 27). The greater improvement in FMA-UE suggests that closed-loop MI-BCI-assisted training may have facilitated motor control at the impairment level, whereas the greater improvement in ARAT indicates that these gains may have extended to task-oriented upper limb activity. This distinction is important because improvement in isolated motor impairment does not necessarily translate into improved object manipulation or functional use of the affected hand. The ARAT findings deserve particular attention because hand-related activities, such as grasping, pinching, and object manipulation, require coordinated distal control, sensorimotor integration, and task-specific movement planning. These functions are often more difficult to recover than more global or proximal upper limb movements. In the present study, the BCI intervention was centered on affected-hand grasping motor imagery and robotic-assisted hand movement. This training structure may have repeatedly paired attempted hand movement with congruent sensory feedback, which may be particularly relevant for patients with limited voluntary movement. By allowing patients to engage motor intention even when overt movement is insufficient, closed-loop MI-BCI-assisted training may help bridge the gap between impaired voluntary output and task-specific sensorimotor practice. However, ARAT is a standardized laboratory-based assessment of upper-limb activity capacity and does not necessarily capture spontaneous use of the affected hand in daily life. Therefore, improved ARAT scores should be interpreted as better performance capacity under structured test conditions rather than as direct evidence of increased real-world use of the paretic arm. Future trials should include real-world upper-limb use measures, such as accelerometry, structured home-use assessments, or patient-reported hand-use scales, to determine whether improvements in activity capacity translate into meaningful daily performance (28–30). The greater improvement in MBI observed in the BCI group may suggest a favorable overall functional trend, but this outcome should be interpreted cautiously. MBI reflects broad basic activities of daily living, including transfers, mobility, self-care, feeding, toileting, and continence. It is influenced not only by upper limb recovery but also by balance, lower limb function, cognition, compensatory strategies, environmental support, and caregiver assistance. Therefore, the MBI result should not be interpreted as direct evidence that the intervention specifically improved upper limb-related independence. Rather, it supports the possibility that improved upper limb motor recovery, when embedded within a comprehensive rehabilitation program, may contribute to broader functional gains. Longer follow-up and more specific measures of upper limb-related daily activity are needed to clarify this relationship.

Cortical reorganization is considered a key neurobiological basis for functional recovery after stroke (31, 32). The therapeutic effects of BCI-based rehabilitation are generally thought to arise from activity-dependent neuroplastic changes within the central nervous system, particularly through closed-loop learning, rather than from peripheral stimulation alone or from nonspecific effects of repeated muscle activation (33, 34). The closed-loop design of MI-BCI training is theoretically compatible with activity-dependent learning models, in which motor intention is temporally paired with contingent sensory feedback. In this study, participants in the BCI group performed affected-hand grasping motor imagery while EEG activity was monitored online; once the individualized EEG control signal reached the predefined threshold, the robotic glove delivered assisted movement with visual, auditory, and tactile feedback. However, Hebbian-like plasticity or neural reorganization was not directly measured, and this interpretation should therefore be regarded as a plausible theoretical explanation rather than a demonstrated causal mechanism. This EEG-contingent feedback may have increased the temporal and functional congruence between internal motor intention and external sensory consequences. Such congruent feedback may enhance attention to the paretic limb, reactivate motor-related cortical networks in the affected hemisphere, and engage brain regions involved in action selection, sensorimotor integration, and multisensory processing (35, 36). Over repeated training sessions, this mechanism may strengthen the sensorimotor loop and promote reorganization of motor and sensory networks within and across hemispheres, particularly through changes in functional connectivity between sensorimotor cortices (37, 38). The exploratory EEG findings provide preliminary neurophysiological support for this interpretation. FFT%α and FFT%β were used in this study as relative spectral power indices during the motor imagery task, reflecting task-related modulation of alpha- and beta-band sensorimotor rhythmic activity. Alpha- and beta-band oscillations are closely involved in motor imagery, movement preparation, sensorimotor integration, and feedback-related motor processing (39–41). The greater changes in FFT%α and FFT%β in the BCI group suggest that closed-loop MI-BCI-assisted training may have altered sensorimotor cortical engagement during motor imagery. These EEG changes are directionally consistent with the observed clinical improvements and may indicate that the intervention influenced neural processes related to motor intention and sensorimotor feedback integration.

This interpretation may also help explain the greater short-term clinical gains observed in the BCI group. Although the control group received comparable robotic assistance and multisensory stimulation, the feedback was not contingent on real-time EEG activity and therefore lacked the specific temporal coupling between motor intention and sensory feedback that characterizes closed-loop MI-BCI training. The additional EEG-contingent closed-loop component may thus have provided a more specific neural training signal, helping to reinforce motor-related cortical engagement and activity-dependent plasticity. This difference may partly explain why the BCI group achieved greater improvements despite comparable training exposure.

The baseline-adjusted ANCOVA results for FFT%α and FFT%β were directionally consistent with the clinical findings, suggesting greater task-related modulation of alpha- and beta-band sensorimotor rhythmic activity during motor imagery in the BCI group. FFT%α and FFT%β were calculated as relative spectral power percentages and were used as exploratory indices of motor imagery-related rhythmic modulation, rather than as direct measures of event-related desynchronization/synchronization, cortical efficiency, or confirmed neuroplastic reorganization. Several mechanisms may plausibly contribute to these neurophysiological changes. In the closed-loop MI-BCI condition, motor imagery of affected-hand grasping was temporally coupled with EEG-contingent robotic assistance and multisensory feedback. This contingency may have strengthened the association between internal motor intention, sensorimotor cortical activation, and external sensory consequences. Repeated pairing of intended movement with congruent visual, auditory, tactile, and proprioceptive feedback may enhance sensorimotor integration, increase attention to the paretic limb, and facilitate more consistent engagement of motor imagery-related networks. In this context, modulation of alpha−/mu- and beta-band activity may reflect changes in processes related to movement preparation, sensorimotor gating, feedback monitoring, and the updating of internal motor representations during repeated training. Nevertheless, these interpretations remain mechanistic hypotheses. Relative alpha- and beta-band power can also be influenced by attentional engagement, fatigue, arousal, task familiarity, and other nonspecific training-related factors. In addition, scalp topographic maps were used only to provide descriptive visualization of frequency-specific EEG activity and should not be interpreted as evidence of confirmed region-specific cortical modulation, because no channel-wise or topographic statistical analysis was performed. Accordingly, the EEG findings should be regarded as exploratory neurophysiological signals that complement the clinical outcomes and provide a rationale for further mechanistic studies. Future research using baseline-normalized ERD/ERS measures, time-frequency methods, source-level analysis, functional connectivity, laterality indices, and clinical-neurophysiological correlation analyses will be needed to determine whether closed-loop MI-BCI training induces specific and durable neuroplastic changes.

The safety and feasibility results were encouraging. No serious adverse events occurred during the 4-week intervention period, and the reported discomforts were mild and transient, suggesting that the training protocol was well tolerated in the randomized sample. The pre-randomization motor imagery familiarization phase helped ensure that participants could understand the task, tolerate the procedures, and engage with the training protocol. Such a run-in procedure is appropriate in an early-stage pilot trial because it can improve adherence and training feasibility, although it also indicates that the present findings are most applicable to patients who are able to complete motor imagery-based training procedures (42, 43). Further studies should evaluate the safety, feasibility, and effectiveness of this intervention in broader stroke populations with different cognitive, attentional, language, and motor imagery capacities.

This study has several methodological strengths. First, the randomized assessor-blinded design helped reduce detection bias and strengthened the rigor of this pilot trial. Second, the use of a dose-matched active control condition allowed a more conservative evaluation of the added value of EEG-contingent closed-loop feedback beyond motor imagery practice, robotic exposure, and multisensory stimulation. Third, the outcome assessment included both impairment-level and activity-level measures of upper limb recovery, providing a clinically relevant multidimensional evaluation. Fourth, the inclusion of exploratory EEG outcomes and scalp topographic visualization provided preliminary mechanistic information linking behavioral recovery with modulation of sensorimotor rhythmic activity.

Several limitations should be acknowledged. First, this single-center pilot study had a relatively small sample size; therefore, the between-group estimates and effect sizes may be unstable and should be interpreted as preliminary rather than definitive evidence of efficacy. The baseline impairment severity was also heterogeneous, and the study was not powered to examine whether treatment response differed by initial motor severity. Second, although the active control design strengthened internal validity, the individual contributions of motor imagery, robotic assistance, multisensory feedback, therapist interaction, and EEG-contingent control cannot be fully separated. The prerandomization motor imagery familiarization phase may also have selected participants who were more able or motivated to engage in motor imagery training, limiting generalizability to broader stroke populations. Third, the 4-week intervention period and absence of follow-up preclude conclusions about durability, particularly during the subacute phase when spontaneous recovery remains active. The lack of objective real-world affected-arm use measures also limits interpretation of whether standardized assessment gains translated into spontaneous daily upper-limb use. Finally, the EEG analyses were exploratory and limited to relative alpha- and beta-band spectral power indices. These findings should not be interpreted as definitive evidence of neural reorganization, cortical efficiency, or region-specific cortical modulation. Future studies should include more comprehensive neurophysiological analyses and clinical-neurophysiological correlation assessments.

In conclusion, this randomized pilot trial indicates that closed-loop MI-BCI-assisted training is feasible and well tolerated in selected patients with subacute stroke. The observed short-term improvements in upper-limb impairment and activity capacity provide preliminary signals of potential benefit beyond dose-matched motor imagery training with non-EEG-contingent feedback. Exploratory EEG findings suggest task-related modulation of alpha- and beta-band sensorimotor rhythmic activity, but should be interpreted as hypothesis-generating rather than confirmatory evidence of neural reorganization. Larger multicenter randomized controlled trials with longer follow-up, rigorous neurophysiological analyses, and real-world upper-limb use outcomes are needed to confirm efficacy, durability, generalizability, and mechanisms.

## Data Availability

The original contributions presented in the study are included in the article/supplementary material, further inquiries can be directed to the corresponding authors.
